# DNA and protein methyltransferases inhibition by adenosine dialdehyde reduces the proliferation and migration of breast and lung cancer cells by downregulating autophagy

**DOI:** 10.1371/journal.pone.0288791

**Published:** 2023-07-28

**Authors:** Rose Ghemrawi, Aya Al Qassem, Azza Ramadan, Raghad Aldulaymi, Nour Sammani, Walaa K. Mousa, Mostafa Khair

**Affiliations:** 1 College of Pharmacy, Al Ain University, Abu Dhabi, United Arab Emirates; 2 AAU Health and Biomedical Research Center, Al Ain University, Abu Dhabi, United Arab Emirates; 3 College of Pharmacy, Mansoura University, Mansoura, Egypt; 4 Core Technology Platforms, New York University Abu Dhabi, Abu Dhabi, United Arab Emirates; University of Florida, UNITED STATES

## Abstract

Protein and DNA methylation is involved in various biological functions such as signal transmission, DNA repair, and gene expression. Abnormal regulation of methyltransferases has been linked to multiple types of cancer, but its link to autophagy and carcinogenesis in breast and lung cancer is not fully understood. We utilized UALCAN, a web tool, to investigate breast and lung cancer database from The Cancer Genome Atlas. We found that 17 methyltransferases are upregulated in breast and/or lung cancer. We investigated the effect of methylation inhibition on two breast cancer cell lines (MDA-MB-231 and MCF-7) and two lung cancer cell lines (H292 and A549) by treating them with the indirect methyltransferase inhibitor adenosine dialdehyde (AdOx). We found that the migration ability of all cell lines was decreased, and the growth rate of MDA-MB-231, MCF-7 and H292 was also decreased after AdOx treatment. These results were correlated with an inhibition of the autophagy in MDA-MB-231, MCF-7 and H292 cell lines, since AdOx treatment induced a decreased expression of ATG7, a reduced ratio LC3-II/LC3-I and an increased p62 level. These findings suggest that inhibiting cells’ methylation ability could be a potential target for breast and lung cancer treatment.

## Introduction

Changes in genes can control the growth and spread of cancer, however, scientists have also recognized the significance of epigenetic regulation in the development of cancer. The importance of histone and DNA methylation in tumorigenesis has been well recognized [1]. For instance, research has linked cancer to overexpression of DNA methyltransferases (DNMTs) [2, 3]. A decrease in DNMT1 has been found to reduce the occurrence of tobacco-induced lung cancer [4]. Increased expression of DNMT3a in lung cancer was linked to the growth and spread of tumor cells [4]. Furthermore, DNMT1, DNMT3a, and DNMT3b were found overexpressed in many breast cancer cell lines such as MDA-MB-231 and MCF-7 [5].

Recent studies have also linked post-translational modifications, notably protein methylation, to carcinogenesis and metastasis [6]. For example, Protein arginine methyltransferases (PRMTs) which add methyl groups to specific nitrogen atoms of arginine, are known to modify histones, to regulate transcription, and to methylate many proteins involved in DNA damage response such as MRE11, Rad 9, 53BP1 and DNA polymerase β, FEN1 [7], so directly influencing cancer development. They also methylate critical proteins in various cancers such as estrogen receptor, BRCA1, EGF, p53 and RNA binding proteins (RBP) [7]. For example, HuR, an RBP, undergoes activation upon methylation by PRMTs, and this activation has been observed to be associated with the development of cancer [8]. Overexpression of PRMTs is often associated with various types of cancer. For example, PRMT1 and PRMT6 promote tumorigenesis and progression of lung, breast and bladder cancer [9, 10], PRMT4 promotes hepatocellular carcinoma [11]. The levels of PRMT5 expression in breast, lung, and human hepatocellular cancer cells are markedly elevated compared to those in normal cells [10, 12]. protein methylation is a significant contributor to the progression of breast and lung cancer, influencing crucial aspects such as DNA damage response and cancer-related signaling pathways. However, the exact mechanisms connecting methylation to the development of cancer remain incompletely comprehended.

Furthermore, autophagy in cancer has been characterized in previous studies, revealing its involvement in both tumor-promoting and tumor-inhibiting roles [13]. Autophagy allows old and damaged cellular material to be degraded by lysosomes, inducing turnover of cell components and providing fresh macromolecular precursors. It was found that once cancers are malignant ones, increased autophagy enables tumor cell survival and growth [14, 15]. Both enhancing and inhibiting autophagy in advanced cancers have been proposed as therapeutic strategies [16, 17], however, the majority of clinical interventions manipulating autophagy in cancer are focusing on inhibiting autophagy [13].

Interestingly, recent research has provided evidence that several tumor suppressor genes and oncogenes associated with autophagy are subject to strict regulation through diverse epigenetic modifications, including DNA methylation and histone modifications [18]. Furthermore, the metabolites of the methyl donor S-adenosylmethionine (SAM) were found to exert important influences on the regulation of autophagy [19]. Consequently, our objective is to investigate the interplay between autophagy and methylation, aiming to unveil novel mechanisms implicated in cancer development and identify potential targets for cancer therapy.

Given that breast and lung cancer currently rank among the most prevalent cancer types globally [20, 21], the discovery of new inhibitory pathways for these specific cancers is of utmost importance. We utilized the UALCAN database, The University of Alabama at Birmingham CANcer data analysis Portal [22], to examine the data of methyltransferases in breast and lung cancer obtained from The Cancer Genome Atlas (TCGA). Our analysis revealed the upregulation of numerous methyltransferases in both types of cancer. Subsequently, we intervened by inhibiting the methylation capacity of breast and lung cancer cell lines to explore the impact on cell proliferation, migration, and autophagy. Specifically, we administered adenosine dialdehyde (AdOx) treatment to two breast cancer cell lines (MDA-MB231, MCF-7) and two lung cancer cell lines (H292, A549). This is a commonly employed broad-spectrum methyltransferase inhibitors [23]. AdOx functions as an inhibitor of AdoHcy (S-adenosyl-L-homocysteine) hydrolase, leading to the accumulation of intracellular AdoHcy levels, the methyltransferase product. This accumulation, in turn, leads to the feedback inhibition of methylation reactions [23]. Furthermore, AdOx is known to inhibit the invasion and growth of various cancer cells [24]. In our study, by analyzing the TCGA database, we indentified 17 methyltransferases upregulated in breast and/or lung cancer. We showed that AdOx treatment decreases autophagy leading to a reduction in MDA-MB231, MCF-7, and H292 cancer cell lines’ growth and migration.

## Materials and methods

### UALCAN

The UALCAN online tool, accessible at http://ualcan.path.uab.edu, utilizes data from the Clinical Proteomic Tumor Analysis Consortium (CPTAC) database and The Cancer Genome Atlas (TCGA) to perform gene expression profiling in both cancer-free and cancerous tissues [22]. The tool was used to analyze the expression of methyltransferases and methylation levels of PRMT1 promoter in breast and lung cancer specimens.

### Cell culture

Human breast (MCF-7 and MDA-MB-231) and lung cancer cell lines (A549 and H292) were used in the present study. All cell lines were purchased from Sigma, MCF-7 (#86012803, Sigma, USA), MDA-MB-231 (#92020424, Sigma, USA), A549 (#86012804, Sigma, USA), H292 (#91091815, Sigma, USA). MCF-7 and MDA-MB-231 were grown in DMEM (#D6429, Sigma, USA), A549 and H292 cells in RPMI-1640 medium (#R8758, Sigma, USA), supplemented with 10% fetal bovine serum (#F9665, Sigma, USA) and antibiotics (#A5955, Sigma, USA) (100 U/ml penicillin and 100 ml/ml streptomycin) in a humidified incubator (5% CO2 in air at 37°C).

### Cell proliferation assay

The colorimetric MTT metabolic activity assay (#ab211091, Abcam, UK) was used to assess cell proliferation. Cancer cell lines (5 × 10^4^ cells/well) were seeded in a 96-well plate at 37°C, and exposed to varying concentrations of AdOx (#A7154, Sigma, USA) or DMSO (#D2650, Sigma, USA) for a duration of 72 hours. The concentrations of AdOx used were 0.01μM, 0.1μM, 1μM, 10μM, and 100μM. Subsequently, the effects of 20μM of AdOx were evaluated at 24, 48, and 72 hours. In addition, A549 cells were treated with concentrations of AdOx ranging from 100μM to 500μM for a duration of 72 hours. After removing the supernatant and washing twice with PBS1X, 20 μl of MTT solution was added to the medium and incubated for 3 hours. Finally, the absorbance intensity was measured by spectrophotometry at the absorbance of 570 nm.

### Wound healing assay

Human cancer cell lines (5 × 10^4^ cells/well) were seeded onto 96-well plates, then treated with either AdOx (#A7154, Sigma, USA) or the vehicle DMSO (#D2650, Sigma, USA). A linear scratch was performed with a 200 μl sterile micropipette tip. Cells were then incubated in a humidified incubator at 37°C and images of the same scratched monolayer were taken after 0, 24, 48 and 72 hours. The images were analyzed using ImageJ software.

### Protein extraction

Cells were treated with 20μM AdOx or the vehicle DMSO for 72 hours. After reaching confluency, live cells were lysed directly with a solution containing 150 mM NaCl, 0.1% sodium dodecyl sulfate, potassium dihydrogen phosphate, 1% Nonidet P40, sodium phosphate anhydrous dibasic, 0.5% sodium deoxycholate, and Complete Protease Inhibitors. Lysates were be subjected to centrifugation at 12000 rpm for 30 min. The supernatant was designated as the cell extract. The protein concentration was determined using BCA Protein Assay kit (#23225, Thermo Fisher Scientific, Waltham, MA, USA) using bovine serum albumin (BSA) as the standard.

### SDS-PAGE and western blot

40 μg of the total protein was loaded per lane of SDS-polyacrylamide gel electrophoresis (PAGE). Proteins from lysates were electro-transferred onto Nitrocellulose membranes, then blocked in 5% skimmed dry milk in Tween-TBS (10 mM Tris-HCl, pH 7.5; 100 mM NaCl; 0.1% Tween-20) for 1 hour. Membranes were then incubated with primary antibodies (1:700); ATG7 (#2631, Cell signaling, USA), LC3A/B (#4108, Cell signaling, USA), SQSTM1/p62 (#5114, Cell signaling, USA), FAK (#3285, Cell signaling, USA), Caspase 3 (#9662, Cell signaling, USA) and GAPDH (#2118, Cell signaling, USA), overnight. After washing them three times in Tween-TBS, they were incubated with secondary antibody (#7074, Cell signaling, USA) (1:2000) for 1 h. Appropriate secondary antibodies conjugated to HRP were used for detection. Quantification was assessed by ImageJ software.

### Statistical analysis

The data provided includes the mean and standard deviation of three measurements. The statistical significance of group analysis was determined by utilizing the student’s test. Statistical analyses were performed using SPSS 18.0 version (SPSS Inc., Chicago, IL, USA). P values less than 0.05 were considered statistically significant. Results were denoted by asterisks in figures (*P < 0.05; **P < 0.01 and ***P < 0.001).

## Results

### Methyltransferases expression in breast and lung cancer

**Fig 1:**
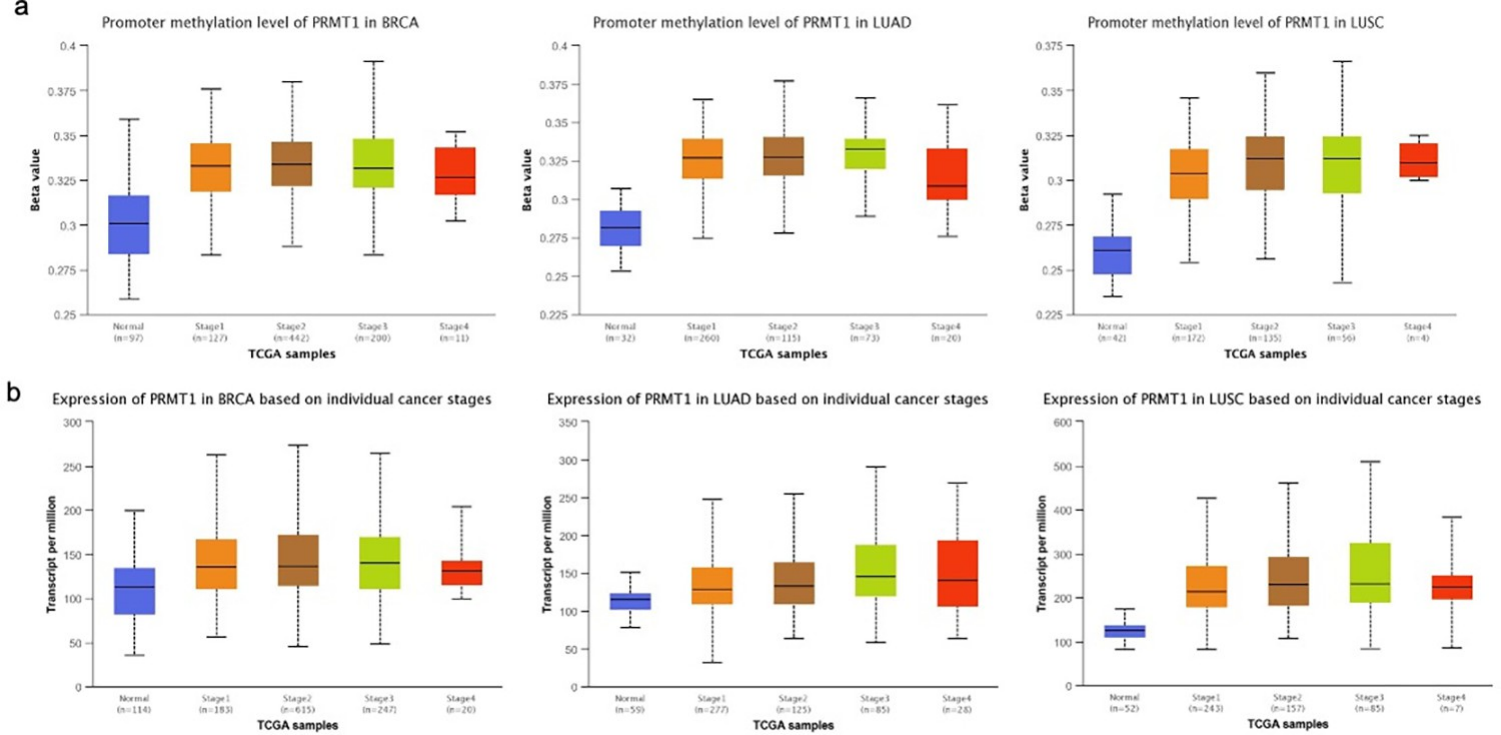
Illustrates PRMT1 expression and methylation level of its promoter in breast and lung cancer specimens. (a) presents the methylation level of PRMT1’s promoter in the aforementioned cancer types. (b) depicts the transcript expression of PRMT1 in different cancer stages (stages 1–4) of breast invasive carcinoma (BRCA), lung adenocarcinoma (LUAD) and lung squamous cell carcinoma (LUSC) groups in comparison with normal group. In this analysis, PRMT1 expression levels were determined using the breast and lung carcinoma database from TCGA through UALCAN.

We utilized UALCAN, a web tool [22] to investigate breast and lung cancer database from The Cancer Genome Atlas (TCGA); link: http://ualcan.path.uab.edu/cgi-bin/. Data at the gene level were used in the analysis. As shown in Table 1, our findings indicate that several methyltransferases are upregulated in breast invasive carcinoma (BRCA), lung adenocarcinoma (LUAD), and lung squamous cell carcinoma (LUSC). Specifically, the following methyltransferases showed upregulation: DNMT1, DNMT3A, DNMT3L, SMYD3, EZH2, PRMT5, and SUV420H2. Additionally, lung cancer demonstrated upregulation of 10 more methyltransferases, namely DNMT3B, SMYD5, EHMT2, SUV39H2, WHSC1, PRDM15, and PRMTs 1, 3, 4, and 6. It is important to note that while the expression of some methyltransferases remains unchanged in cancer, alterations in the methylation profile of their promoters are observed. As an example, our analysis results highlight the significant increase in the methylation level of PRMT1’s promoter in BRCA, LUAD, and LUSC compared to the control groups (P<0.05) (Fig 1A), regardless of the cancer stage, even though the gene was significantly upregulated in LUSC only (Fig 1B).

**Table 1:** Explores methyltransferase expression levels using the breast and lung carcinoma database from TCGA through UALCAN. Link: http://ualcan.path.uab.edu/cgi-bin/. DNMT: DNA methyltransferases; SMYD: Su(Var)3–9, Enhancer-of-zeste and Trithorax (SET) and Myeloid, Nervy, and DEAF-1 (MYND) domain-containing; PRMT: Protein arginine methyltransferase; EZH: Enhancer of zeste homolog; EHMT: Euchromatic histone-lysine N-methyltransferase; SUV39H: Short for suppressor of variegation 3(9) homologue; WHSC1: Wolf-Hirschhorn syndrome candidate gene-1; SUV420H: Suv4-20h histone methyltransferase; PRDM: PRDF1 and RIZ1 homology domain methyltransferase.

Gene	Gene expression in Breast adenocarcinoma	Gene expression in Lung adenocarcinoma	Gene expression in Lung squamous cell carcinoma
DNMT1	**Upregulated**	**Upregulated**	**Upregulated**
DNMT3A	**Upregulated**	**Upregulated**	**Upregulated**
DNMT3B	Insignificant change	**Upregulated**	**Upregulated**
DNMT3L	**Upregulated**	**Upregulated**	**Upregulated**
SMYD1	*Downregulated*	Insignificant change	Insignificant change
SMYD2	Insignificant change	Insignificant change	Insignificant change
SMYD3	**Upregulated**	**Upregulated**	Insignificant change
SMYD4	*Downregulated*	Insignificant change	Insignificant change
SMYD5	Insignificant change	**Upregulated**	**Upregulated**
PRMT1	Insignificant change	Insignificant change	**Upregulated**
PRMT2	Insignificant change	Insignificant change	Insignificant change
PRMT3	Insignificant change	**Upregulated**	**Upregulated**
PRMT4	Insignificant change	**Upregulated**	**Upregulated**
PRMT5	**Upregulated**	**Upregulated**	**Upregulated**
PRMT6	Insignificant change	Insignificant change	**Upregulated**
PRMT7	Insignificant change	Insignificant change	Insignificant change
PRMT8	Insignificant change	*Downregulated*	*Downregulated*
EZH1	*Downregulated*	Insignificant change	*Downregulated*
EZH2	**Upregulated**	**Upregulated**	**Upregulated**
EHMT1	Insignificant change	Insignificant change	Insignificant change
EHMT2	Insignificant change	**Upregulated**	**Upregulated**
SUV39H1	Insignificant change	Insignificant change	**Upregulated**
SUV39H2	Insignificant change	**Upregulated**	**Upregulated**
WHSC1	Insignificant change	**Upregulated**	**Upregulated**
SUV420H1	Insignificant change	Insignificant change	Insignificant change
SUV420H2	**Upregulated**	**Upregulated**	**Upregulated**
PRDM1-4,7–10,12–14	Insignificant change	Insignificant change	Insignificant change
PRDM5	*Downregulated*	*Downregulated*	*Downregulated*
PRDM11	*Downregulated*	Insignificant change	Insignificant change
PRDM15	Insignificant change	**Upregulated**	**Upregulated**
PRDM16	Insignificant change	*Downregulated*	*Downregulated*

### Reduced proliferation rate of breast and lung cancer cell lines after treatment with the methyltransferase inhibitor AdOx

**Fig 2:**
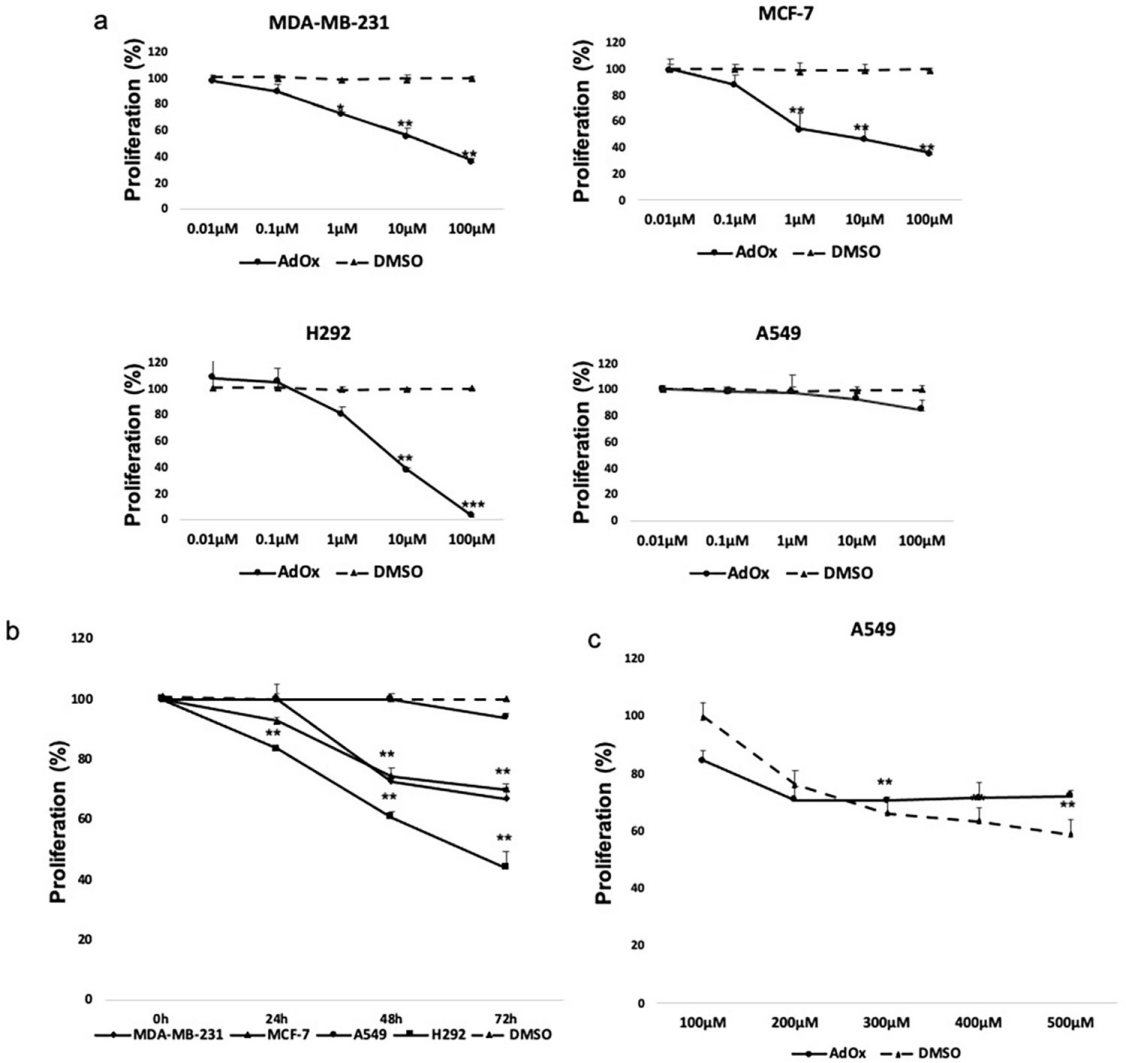
The effect of AdOx treatment on the proliferation of breast and lung cancer cell lines. (a) Breast and lung cancer cell lines were treated with different concentrations (0.01μM, 0.1μM, 1μM, 10μM and 100μM) of AdOx (and DMSO as control) for 72h and cell proliferation was analyzed by MTT assay. (b) The percentage of proliferation of MCF-7, MDA-MB-231, H292, A549 cell lines treated or not with 20 μM AdOx determined by MTT assay at 24, 48 and 72h. (c) A549 cell line was treated with different concentrations (100μM, 200μM, 300μM, 400μM and 500μM) of AdOx (and DMSO as control) for 72h and cell proliferation was analyzed by MTT assay. Significant results were denoted by asterisks (*P < 0.05; **P < 0.01).

Due to the upregulation of methyltransferases in breast and lung cancer, along with the high methylation levels observed in some of their promoters, we recognized the potential promise of investigating the impact of methylation inhibition in these types of cancer. Consequently, we embarked on assessing the effect of inhibiting methylation on tumor proliferation in breast and lung cancer. The methyltransferase inhibitor AdOx has been shown to hinder the growth of various cancer cells due to its transmethylation blocking ability [24–26]. In this study, the proliferation percentages of four cancer cell lines (MCF-7, MDA-MB-231, H292, A549) were first analyzed in presence and absence of different concentrations (0.01μM, 0.1μM, 1μM, 10μM and 100μM) of AdOx and DMSO (as control) for 72h. It was found that AdOx significantly inhibited the proliferation of MCF-7, MDA-MB-231, H292 in a dose dependent manner without affecting A549 cell lines (Fig 2A). Then we examined the effect of 20μM AdOx [27] on cells’ proliferation after 24h, 48h and 72h of treatment (Fig 2B). Our investigation revealed that the concentration of 20μM demonstrated notable efficacy in significantly inhibiting the proliferation of all tested cancer cell lines except A549. This effect was observed after 24 hours of treatment for H292 cells and after 48 hours for breast cancer cell lines. To determine the cytotoxic concentration of AdOx on the A549 cell line, we conducted an MTT assay. A549 cells were treated with various concentrations of AdOx (100μM, 200μM, 300μM, 400μM, and 500μM) for a duration of 72 hours. As shown in Fig 2C, our findings revealed that subjecting A549 cells to elevated concentrations of DMSO treatment induced cytotoxicity. However, we did not observe a substantial impact of AdOx on cell proliferation in this particular cell line.

### Migration ability of breast and lung cancer cell lines decreases after AdOx treatment

**Fig 3:**
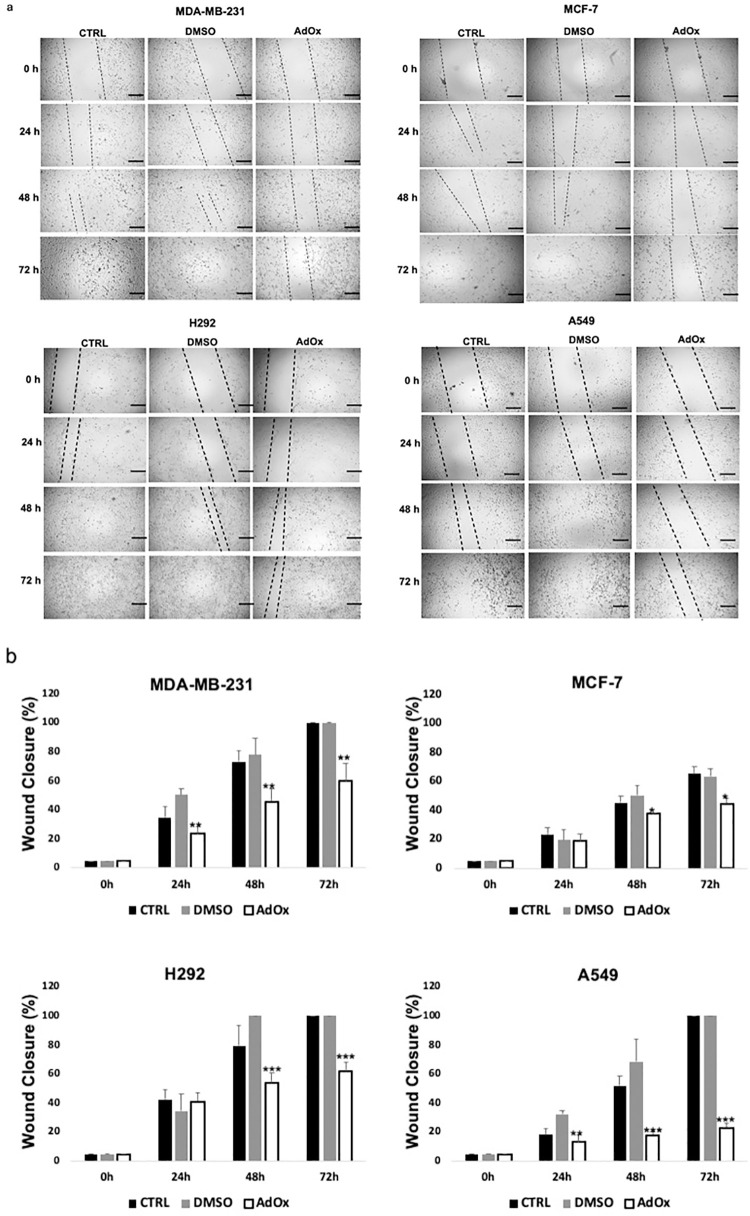
Reduced migration ability of breast and lung cancer cell lines after AdOx treatment. (a) Representative images from wound healing assay of MDA-MB-231, MCF-7, H292 and A549 treated with 20μM AdOx or the vehicle (DMSO) for 24, 48 and 72h. (b) The percentage of the wound closure covered by migrated cells was quantified and shown as the mean ± SD of three independent experiments. The data demonstrates a statistically significant reduction in migration for AdOx-treated cells compared to the cells treated with the control, DMSO. The scale bar is 400 μm. Significant results were denoted by asterisks (*P < 0.05; **P < 0.01 and ***P < 0.001).

AdOx treatment has been shown to have a suppressive effect on tumor cell migration and invasion, as reported in previous studies [25, 26]. Therefore, we evaluated the impact of 20μM AdOx for 24, 48 and 72h on the migration of cancer cell lines by performing a wound healing assay. As shown in Fig 3A, the treatment with 20μM of AdOx resulted in a time-dependent decrease in the migration capability of all the tested cancer cell lines. Subsequently, we compared the migrated area of cells treated with AdOx to that of cells treated with the control (DMSO). Our analysis revealed a significant reduction in the migrated area of MDA-MB-231, MCF-7, H292, and A549 cells following AdOx treatment, in comparison to the control group (Fig 3B). Following treatment, the MDA-MB-231 cells showed a wound healing percentage of 23.6% after 24 hours, 45.9% after 48 hours, and 60% after 72 hours. Similarly, the MCF-7 cells exhibited a wound closure percentage of 18.9% after 24 hours, 37.7% after 48 hours, and 44.5% after 72 hours. In terms of migration activity, the H292 cell line demonstrated 41% migration after 24 hours and 53.8% after 48 hours, while the A549 cell line showed 13.5% migration after 24 hours and 18.1% after 48 hours. After 72 hours, the migration activity increased to 61.7% for H292 cells and 23.2% for A549 cells. Statistical analysis of the data revealed a significant decrease in migration when comparing the cells treated with AdOx to those treated with the control, DMSO.

### Autophagy downregulation in breast and lung cancer cell lines after AdOx treatment

**Fig 4:**
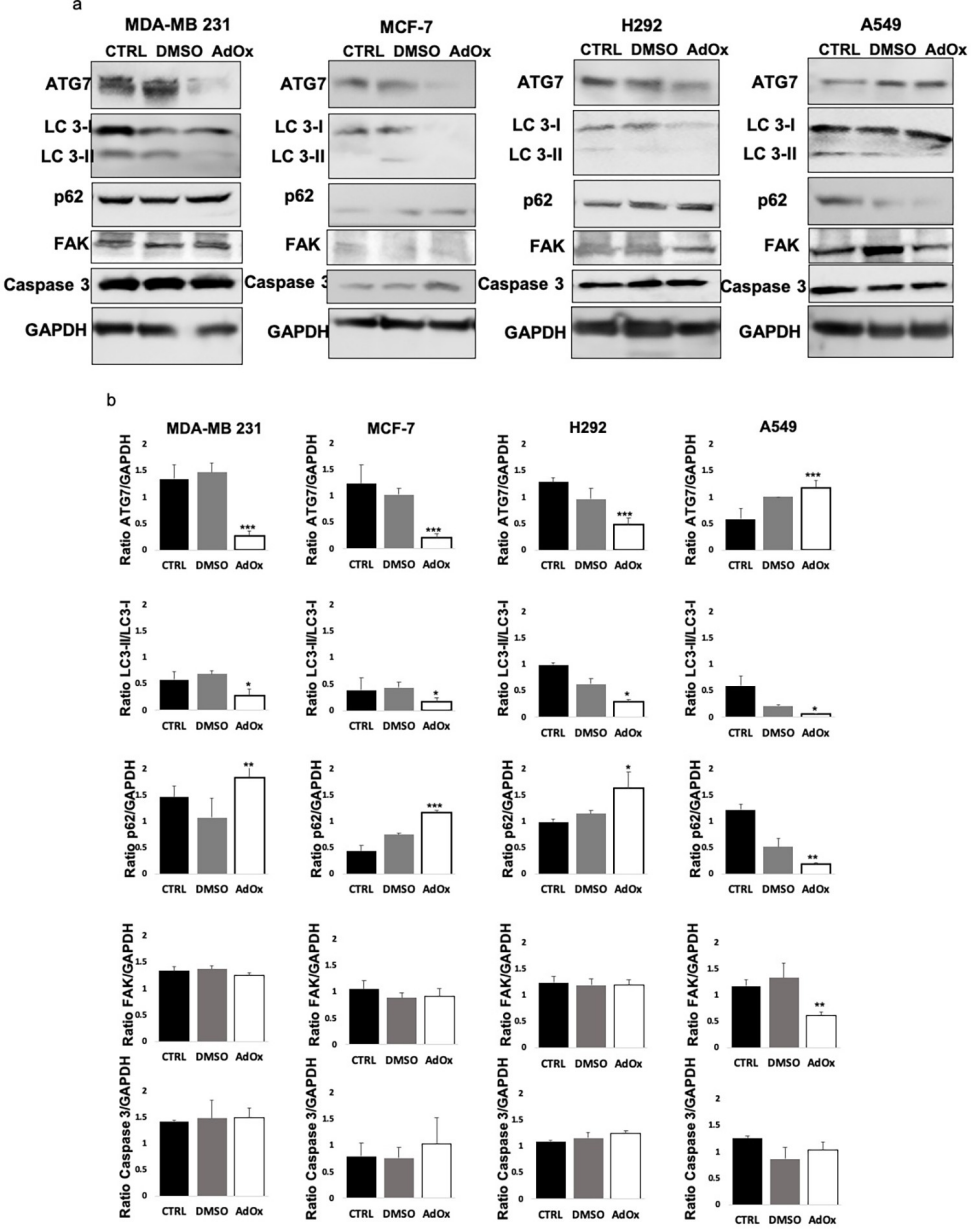
Autophagy inhibition in MDA-MB-231, MCF-7 and H292 cancer cell lines after AdOx treatment. (a) Western blots of ATG7, LC3, p62, FAK, Caspase 3 and GAPDH as control in cancer cell lines treated with 20μM AdOx for 72h versus the untreated control (CTRL) or treated with DMSO. (b) The protein expression was quantified using ImageJ and shown as the mean ± SD of three independent experiments. The data clearly shows a significant decrease in ATG7 expression, the ratio of LC3-II to LC3-I, and an increase in p62 levels in AdOx-treated MDA-MB-231, MCF-7, and H292 cells compared to the control cells treated with DMSO. Significant results were denoted by asterisks (*P < 0.05; **P < 0.01 and ***P < 0.001). Original blots are shown in S1 Raw images.

To elucidate the mechanism underlying the observed decrease in cancer cell proliferation and migration upon methylation inhibition, we conducted investigations into the role of autophagy. We examined the expression of the proteins ATG7 (Autophagy Related 7) known to be an essential autophagy effector enzyme, LC3 (Light Chain 3 isoforms) known to switch from LC3-I to LC3-II through a ubiquitin-like system involving ATG7, which allows LC3 to associate with autophagic vesicles, and p62 (or SQSTM1) which binds autophagosome membrane proteins, bringing p62-containing protein aggregates to the autophagosome. Therefore, an increased expression of ATG7 and LC3-II to LC3-I ratio, and a decreased p62 is correlated with autophagy activation. As shown in Fig 4 and S1 Raw images, treating MDA-MB-231, MCF-7 and H292 cells lines with 20μM AdOx for 72h reduced the expression of ATG7 and the ratio of LC3-II to LC3-I, and increased the expression of p62, showing that AdOx inhibited the autophagy of the three cancer cell lines. Notably, in A549 cell lines, although AdOx treatment resulted in a decrease in the LC3-II to LC3-I ratio, it paradoxically led to an increase in the expression of ATG7 and a decrease in the expression of p62.

To validate that the observed decline in cancer cell proliferation and migration in MDA-MB-231, MCF-7, and H292 cells was primarily attributable to the inhibition of autophagy rather than apoptosis, we investigated the expression of the biomarker: caspase 3. As shown in Fig 4, treatment with AdOx did not result in any noteworthy changes in the expression of caspase 3 in any tested cell line. Consequently, the reduction in proliferation observed is not dependent on apoptosis. Interestingly, assessing the expression of the migration marker FAK [28] showed a significant reduction in its expression only in A549 cells treated with AdOx (Fig 4). This finding provides an explanation for the effect of AdOx on the observed reduction in migration capacity in this particular cell line, while not affecting autophagy and proliferation.

## Discussion

Protein and DNA methylation occurs in various cell types, from prokaryotes to eukaryotes, and recent advancements in epigenetic research have highlighted its importance. Histone protein methylation is a dynamic process that contributes to the epigenetic histone code, and it is closely regulated by histone methyltransferases and demethylases, which are essential for controlling gene transcription. Additionally, many nonhistone proteins can also undergo methylation, which impacts various cellular functions such as RNA processing, translation, signal transduction, DNA damage response, and cell cycle regulation [29]. Protein methylation has recently been recognized as crucial in regulating cell cycle and DNA repair processes. As protein methylation dysregulation is strongly associated with cancer development, protein methyltransferases are a promising target for developing anticancer drugs. For example, several PRMT inhibitors are undergoing phase 1/2 clinical trials [30]. Furthermore, certain methylation markers, such as DAL-1, EPHB6, HS3ST2, TMEM88 and MGMT, specifically associated with lung cancer progression and metastasis, were found to have increased methylation levels [31]. Also, previous research revealed that methylation of the BRCA1 promoter in peripheral blood DNA was linked to a 3.5-fold increased risk (95% CI, 1.4–10.5) of developing breast cancer before the age of 40 [32, 33]. Another study conducted by Hansmann et al. found that 1.4% of 600 women in the German Consortium for Hereditary Breast and Ovarian Cancer exhibited constitutive hypermethylation of the BRCA1 gene [33, 34]. Furthermore, hypermethylation of the ATM gene (Ataxia Telangiectasia Mutated gene) body was associated with a threefold higher risk of developing breast cancer [33, 35].

In our study, we found that breast and lung cancer cells exhibit elevated levels of numerous methyltransferases when compared to normal cells. Specifically, we observed the upregulation of seven methyltransferases in breast cancer and seventeen in lung cancer. Additionally, it was intriguing to note that while the expression of certain methyltransferases remained unchanged in cancer cells, alterations in the methylation profile of their promoters were evident. These findings strongly suggest the potential of targeting methyltransferases by inhibiting the methylation process within cancer cells. Consequently, we embarked on this study with the objective of evaluating the impact of methylation inhibition on the proliferation and migration of breast and lung cancer cells, utilizing AdOx as an indirect methyltransferase inhibitor.

Not many previous studies assessed the effect of AdOx in cancer. However, the use of AdOx has been shown to decrease cancer cell survival and induce apoptosis [23–25]. It was found to promote cellular senescence in breast cancer [36], to reduce the expression of MMP-9 and limit the invasion of human glioblastoma and breast cancer cell lines via inhibiting the Ras/Raf-1/ERK/AP-1 pathway [24]. Also, AdOx exhibited cytotoxic properties against androgen-dependent, androgen-sensitive, and castration-resistant prostate cancer cells in laboratory settings [25], and it was able to suppress the growth of prostate cancer in an in vivo mouse xenograft model [25]. In head and neck cancer, after administering AdOx, a decrease in asymmetric dimethylarginine (ADMA) levels was observed and the growth rate of cells significantly decreased [26]. In our study, we observed a considerable reduction in proliferation among breast cancer cell lines and the lung cancer cell line H292 following a 72-hour treatment with various concentrations of AdOx. However, in the case of the lung cancer cell line A549, treatment with the same concentrations of AdOx only resulted in a slight decrease in the growth rate. Interestingly, a prior study examining the DNA methylome profiling of A549 cells indicated that the DNA methylation of this particular cell line cannot be readily altered within a short timeframe [37]. This observation implies that the methylation level of A549 cells might be resistant to the influence of AdOx, and it is possible that its initial methylation level was already comparatively low when compared to MDA-MB-231, MCF-7, and H292 cell lines. Consequently, the treatment with AdOx appears to be more effective in suppressing the growth of the latter three cell lines (MDA-MB-231, MCF-7, and H292) in comparison to A549 cells.

Our study showed the ability of AdOx treatment to significantly diminish the migration activity of breast and lung cancer cells. We observed a marked reduction in the migration ability of the tested cancer cell lines, namely MDA-MB-231, MCF-7, H292, and A549, following treatment with AdOx. This suggests that AdOx has the potential to not only impede the growth but also inhibit the migration of breast and lung cancer cells Previous studies have also documented the inhibitory effects of AdOx treatment on the migration and invasion of tumor cells [26, 27].

In order to gain understanding about how AdOx suppresses tumors and to depict the mechanism leading to the inhibition of proliferation and migration of cancer cells, we examined the effect of methylation inhibition on autophagy. Interestingly, we found that the treatment with AdOx in MDA-MB-231, MCF-7, and H292 cell lines resulted in the inhibition of autophagy. This was evidenced by a decrease in ATG7 expression and a reduction in the ratio of LC3-II to LC3-I, along with an increase in p62 levels. ATG7 and LC3-II are known to be important in the formation of autophagosome [38]. p62 is a widely recognized autophagic protein that has the ability to attach to the autophagic receptor protein ATG8/LC3, thus influencing the process of cell autophagy [39]. The link between methylation inhibition and reduced autophagy can be explained by a previous study which demonstrated that the RNA binding protein HuR regulates the expression of autophagy-related proteins. This regulation occurs through the binding of HuR to the AU-rich element of ATG7’s mRNA, thereby influencing its stability [40]. Interestingly, it has been observed that the activity of HuR is regulated by methylation. Specifically, a decrease in methylation levels has been shown to lead to a reduction in HuR’s activity [41].

The impact of AdOx treatment on autophagy in A549 cell lines exhibited conflicting results. Specifically, AdOx treatment resulted in an increase in the expression of ATG7, a decrease in p62 levels, and a decrease in the ratio of LC3-II to LC3-I. These findings do not allow for a definitive conclusion regarding the effect of AdOx on autophagy in A549 cells. The variation in results among the cell lines is not unexpected, as previous studies have demonstrated that autophagy can be either upregulated or downregulated in cancer cells depending on the specific tumor type and stage [42]. Autophagy is known to play a dual role in cancer, both promoting and suppressing tumor growth. It serves as an inhibitor during tumor initiation and malignant transformation by eliminating damaged cells and organelles, as stated in previous research [43]. Also, it has a protective function during cancer development by fulfilling the metabolic and energy production requirements of cancer cells [43].

We examined the expression of caspase 3 in cells treated with AdOx, and we observed that there was no significant alteration in caspase 3 levels following the treatment. This finding suggests that the reduction in cell proliferation induced by AdOx is not dependent on apoptosis via caspase 3 activity, and instead, it appears to be associated with the inhibition of autophagy in the MDA-MB-231, MCF-7, and H292 cell lines. These results contradict a previous study that demonstrated AdOx- growth suppression in prostate cancer cells through the inhibition of apoptosis [25]. Therefore, further investigation is needed to explore the effects of AdOx on apoptosis in lung and breast cancer cell lines.

From our findings, we can deduce that the impact of AdOx on the MDA-MB-231, MCF-7, and H292 cell lines differs from its effect on A549. AdOx did not influence the proliferation of the A549 cell line and did not exhibit a clear effect on autophagy. However, it did lead to a decrease in the migratory capability. Interestingly, this reduction was accompanied by a decline in the expression of the cell migration marker FAK [28] specifically in A549 cells treated with AdOx, whereas no such effect on FAK expression was observed in the other three cell lines. The relationship between the inhibition of methyltransferase and FAK has not been previously extensively explored.

The present study thus provides fundamental background for future evaluation of using AdOx for breast and lung cancer treatment. Further application of methylation inhibition in therapeutic use should require analyses of methyltransferases levels in breast and lung cancer cell lines, in animal models and in pathological cancer specimens. Furthermore, developing novel inhibitors targeting specific methyltransferases implicated in cancer is crucial to enable precise modulation of aberrant methylation patterns in cancer cells. In conclusion, the inhibition of methyltransferases represents an exciting avenue in cancer research with significant future prospects. Continued research and clinical investigations will advance our understanding of these inhibitors, allowing for their successful translation into effective therapies for various cancer types.

## Supporting information

S1 Raw images Original blots. (PDF)

S1 Graphical abstract (DOCX)

## Abbreviations

53BP1: p53-binding protein 1
AdOx: Adenosine dialdehyde
AP-1: Activator protein-1
ATM: Ataxia telangiectasia mutated gene
BRCA: Breast invasive carcinoma
BRCA1: Breast cancer type 1 susceptibility protein
DAL-1: Differentially expressed in adenocarcinoma of the lung
DMSO: Dimethyl sulfoxide
DNMT: DNA methyltransferases
EGF: Epidermal growth factor
EHMT: Euchromatic histone-lysine N-methyltransferase
EPHB6: Eph receptor tyrosine kinase B6
ERK: Extracellular signal-regulated kinase
EZH: Enhancer of zeste homolog methyltrasnferase
FAK: Focal adhesion kinase
FEN1: Flap endonuclease 1
HS3ST2: Heparan sulfate 3-O-sulfotransferase 2
HuR: Human antigen R
LUAD: Lung adenocarcinoma
LUSC: Lung squamous cell carcinoma
MGMT: O^6-Methylguanine-DNA methyltransferase
MMP-9: Matrix metalloproteinase-9
MRE11: Meiotic recombination 11
MTT: 3-(4,5-dimethylthiazol-2-yl)-2,5-diphenyl tetrazolium bromide
PBS: Phosphate buffer saline
PRDM: PRDF1 and RIZ1 homology domain methyltransferase
PRMT: Protein arginine methyltransferase
PRMT: Protein arginine methyltransferase
Rad 9: Radiation sensitive 9
Ras: Rat sarcoma
RBP: RNA binding protein
SAM: S-adenosylmethionine
SMYD: Set and MYND domain-containing protein
SUV39H: Short for suppressor of variegation 3(9) homologue
SUV420H: Suv4-20h histone methyltransferase
TMEM88: Transmembrane protein 88
WHSC1: Wolf-Hirschhorn syndrome candidate gene-1
