# Supplementary figures and images for: DNA and protein methyltransferases inhibition by adenosine dialdehyde reduces the proliferation and migration of breast and lung cancer cells by downregulating autophagy
Source: PLoS One. 2023 Jul 28;18(7):e0288791. doi: 10.1371/journal.pone.0288791 (PMC10381035; doi:10.1371/journal.pone.0288791)

S1\_raw\_images. Original blots.

## MDA-MB 231

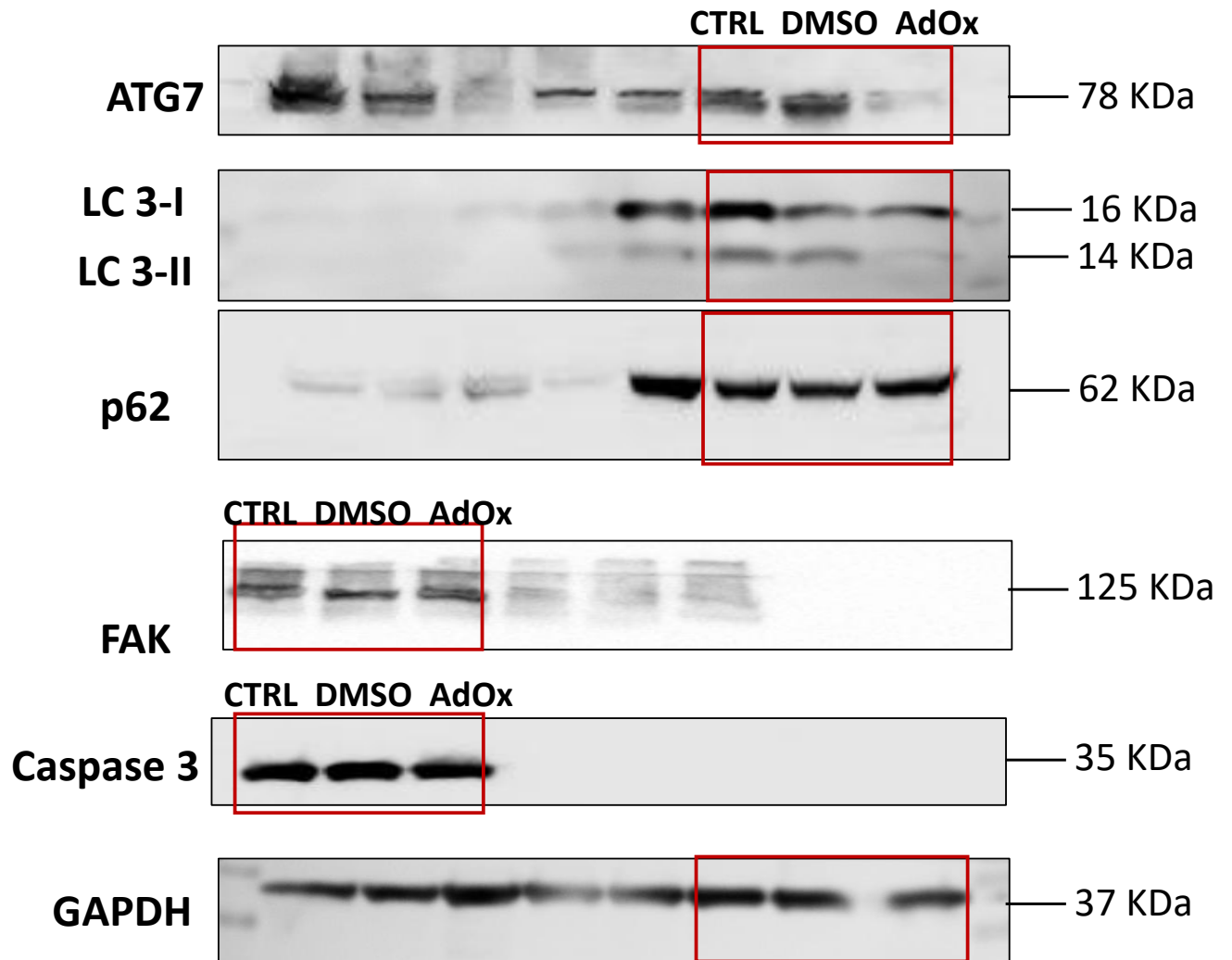

# MCF-7

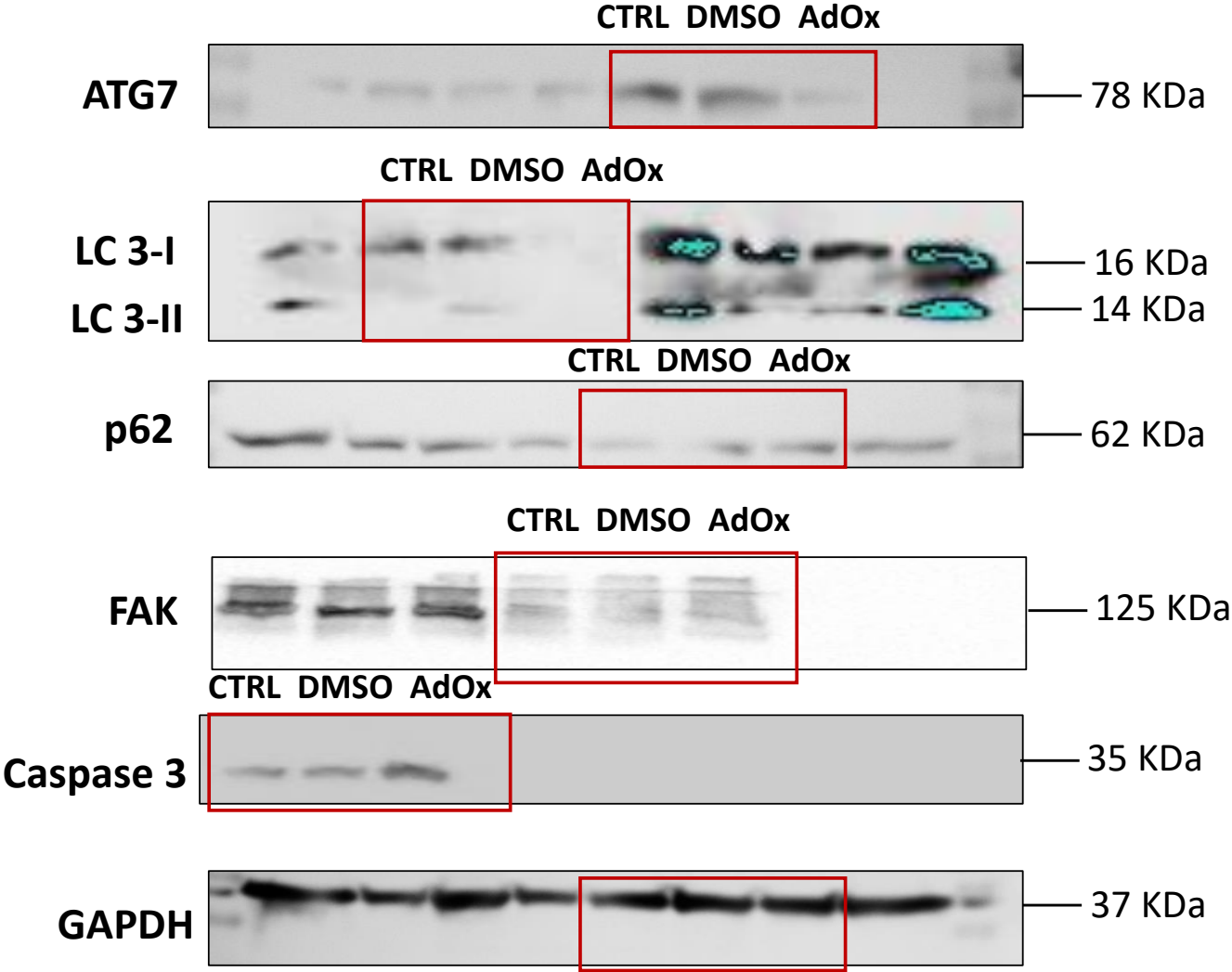

# H292

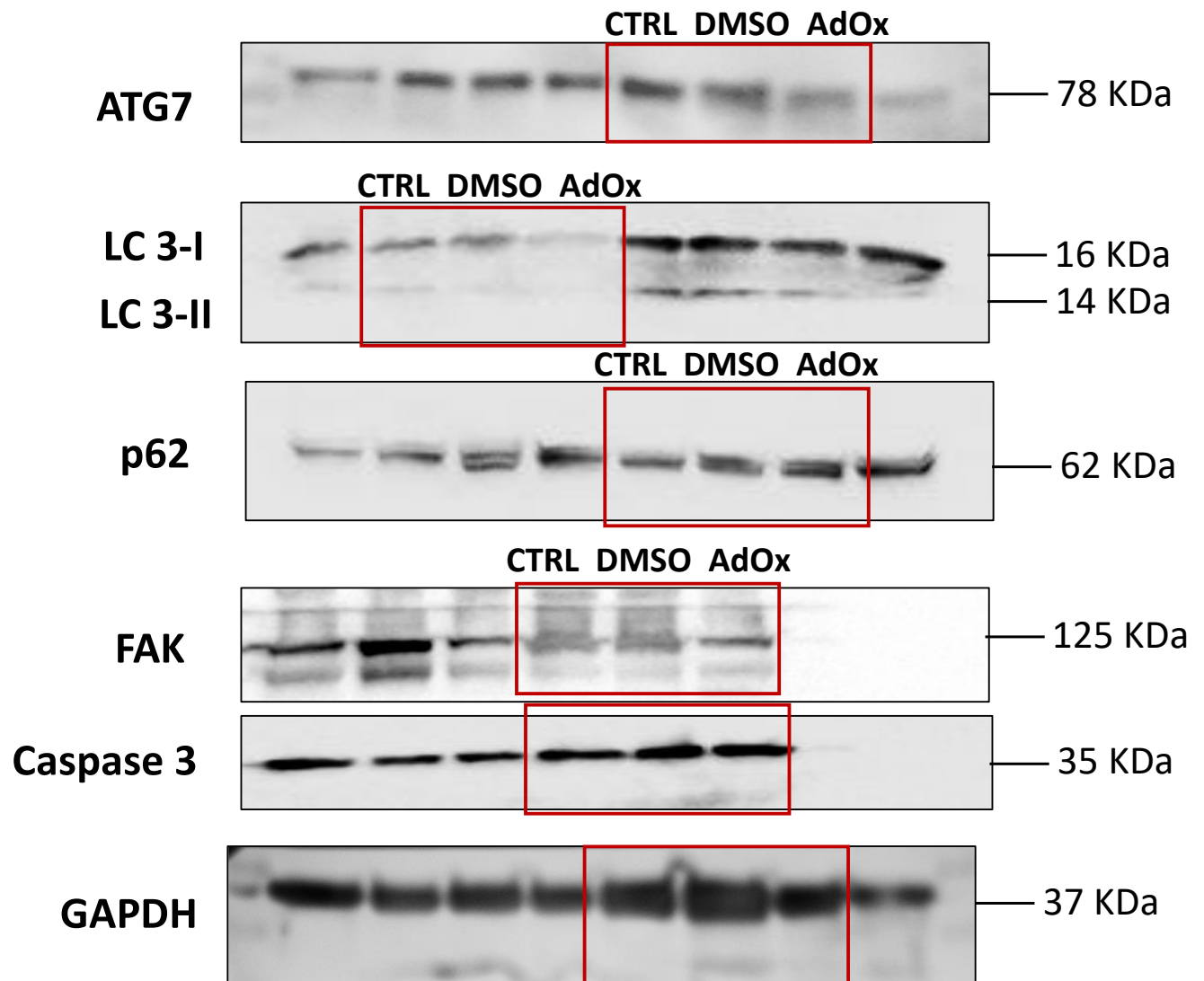

# A549

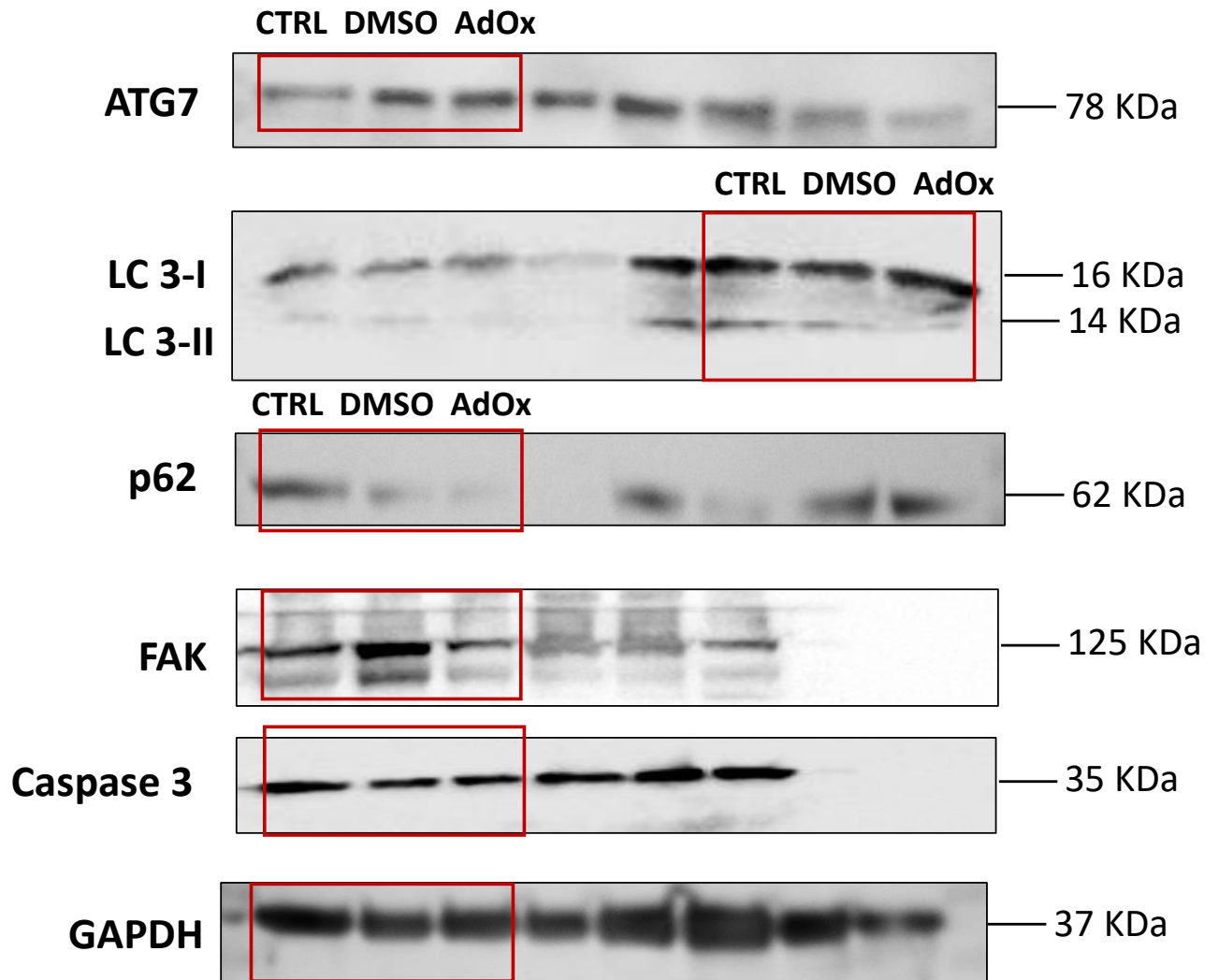

Supplement: S1 Raw images — (PDF) [file pone.0288791.s001.pdf]

**Graphical abstract:**

**
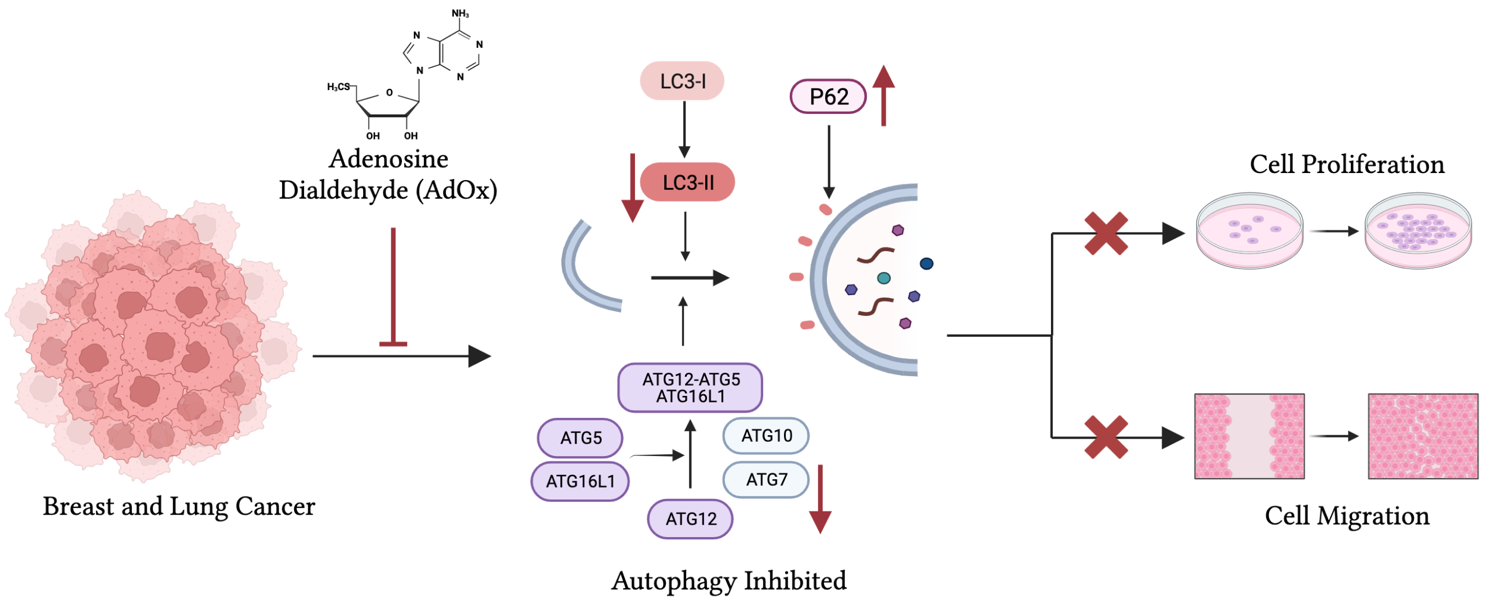
**

Supplement: S1 Graphical abstract — (DOCX) [file pone.0288791.s002.docx]
